# Utilization of a 3D printer to fabricate boluses used for electron therapy of skin lesions of the eye canthi

**DOI:** 10.1002/acm2.12013

**Published:** 2016-11-30

**Authors:** Magdalena Łukowiak, Karolina Jezierska, Marek Boehlke, Marzena Więcko, Adam Łukowiak, Wojciech Podraza, Mirosław Lewocki, Bartłomiej Masojć, Michał Falco

**Affiliations:** ^1^ Department of Medical Physics West Pomeranian Oncology Center Szczecin Poland; ^2^ Department of Medical Physics Pomeranian Medical University Szczecin Poland; ^3^ Department of Medical Devices Samodzielny Publiczny Wojewódzki Szpital Zespolony im. Marii Skłodowskiej–Curie Szczecin Poland; ^4^ Department of Radiotherapy West Pomeranian Oncology Center Szczecin Poland

**Keywords:** 3D printing, bolus, teleradiotherapy

## Abstract

This work describes the use of 3D printing technology to create individualized boluses for patients treated with electron beam therapy for skin lesions of the eye canthi. It aimed to demonstrate the effectiveness of 3D‐printed over manually fabricated paraffin boluses. The study involved 11 patients for whom the construction of individual boluses were required. CT scans of the fabricated 3D‐printed boluses and paraffin boluses were acquired and superimposed onto patient CT scans to compare their fitting, bolus homogeneity, and underlying dose distribution. To quantify the level of matching, multiple metrics were utilized. Matching Level Index (ML) values ranged from 0 to 100%, where 100% indicated a perfect fit between the reference bolus (planned in treatment planning system) and 3D‐printed and paraffin bolus. The average ML (± 1 SD) of the 3D‐printed boluses was 95.1 ± 2.1%, compared to 46.0 ± 10.1% for the manually fabricated paraffin bolus. Correspondingly, mean doses were closer to the prescribed doses, and dose spreads were less for the dose distributions from the 3D‐printed boluses, as compared to those for the manually fabricated paraffin boluses. It was concluded that 3D‐printing technology is a viable method for fabricating boluses for small eye lesions and provides boluses superior to our boluses manually fabricated from paraffin sheets.

## 1 Introduction

Radiotherapy using electron beams, for example Electron beam therapy (EBT) is used in the radiotherapy treatment of neoplastic changes located on or near the surface of the skin. One example of these neoplastic changes is skin lesions of the eye canthus, which are usually basal cell or more rarely squamous cell in character.^1^ The location of the tumor lesion in this region is particularly disadvantageous, because after penetration of the tumor into the eye socket, further spread can be almost symptomless.^2^ There are two main methods for treating skin lesions of the eye canthus — radiotherapy and surgical excision. However, physicians more often choose radiotherapy than extensive surgery,^3^, ^4^, ^5^ as radiotherapy can achieve excellent results in terms of local control, cosmetics, and functionality.^6^, ^7^ EBT is typically used, due to the limited extent to which the beams penetrate, leaving healthy tissues distal to the planning target volume (PTV) protected, while delivering an acceptably uniform dose distribution to the PTV.

According to the International Commission on Radiation Units and Measurements (ICRU) Report 71, the dose distribution should result in complete PTV coverage by the 90% (of assigned dose) dose surface.^8^ A homogeneous dose distribution in the PTV is affected by two factors: (1) the location of the tumor and (2) the curvature of the surface through which the electron beam enters. The closer the neoplastic change to the skin, the higher the possibility that dose distribution in the area of change may be insufficient, typically due to the phenomenon of dose build‐up. As mentioned, the curvature of the surface (through which the beam enters) can also cause heterogeneity in the dose distribution. This situation results from an uneven scattering of electrons due to a change in distance between the source and irradiated surfaces.

The potential negative impact of each of the aforementioned factors on the desired dose distribution can be reduced by the use of an individualized bolus, which is a tissue‐like material leveling the surface of the patient to be approximately perpendicular to central axis. Such a bolus increases skin dose, homogenizes dose heterogeneity resulting from the irregular skin surface, and protects distal tissues by conforming dose line to the distal PTV surface.^9^ Similarly, bolus electron conformal therapy (ECT) has been shown clinically useful for many clinical sites^10^ such as chest wall irradiation (postmastectomy irradiation),^11^, ^12^, ^13^ head and neck radiotherapy,^14^, ^15^, ^16^, ^17^, ^18^, ^19^, ^20^, ^21^, ^22^, ^23^ paraspinal muscle region,^24^ and extremities.^25^

Most of the published papers describe larger PTVs using electron fields with the bolus having a shaped upstream surface; however, papers describing bolus ECT of the nose^26^ are similar to the present case of eye canthi, where bolus is used primarily to remove dose heterogeneity due to the irregular surface of the nose.

A number of factors are considered when designing the bolus; most notably, the ability of the electrons to correctly penetrate to depth at which the target is located. The shape, size, and thickness (height) of the bolus is designed in the treatment planning system (TPS), based on computed tomography of the patient, as a separate structure having a density equal to the average density of the soft tissue, 1 g/cm^3^. The next step, the production of a bolus designed in the TPS, is the most difficult and the most crucial in the process. One of the methods of bolus production involves hand molding on the patient — a method vitiated by the very fact that pressing individual layers of paraffin or polymer bolus directly on the patient's skin will affect the shape of the bolus and dose distribution in relation to the reference model (planned in TPS). Another methods of bolus production is using milled electron bolus technology, being fabricated using computer‐controlled milling machines.^9^, ^13^, ^22^, ^24^ Although milled bolus ECT technology is the only commercially available design and fabrication technology (decimal LLC, Sanford, FL, USA), its availability is largely limited to the United States of America.

Recently, there has been increased interest in investigating three‐dimensional (3D) printing technologies to produce patient‐specific objects for use in a medical context, including boluses in radiotherapy.^9^, ^10^, ^11^, ^12^, ^13^, ^22^, ^23^, ^24^, ^25^, ^26^, ^27^, ^28^, ^29^, ^30^, ^31^ Although 3D printing technology offers a viable technology for small boluses designed by individual clinics, its clinical use in radiotherapy is relatively recent. One reason for this is the difficulty in obtaining a medical certificate for materials used in 3D printers, which involves obtaining the agreement of the ethics committee and consent of the patient, as contact between the printed bolus and the surface of the patient's body. Furthermore, there is a lack of worldwide commercial availability of bolus design tools, such as those reported by Low et al.^31^ and Su et al.,^25^ which are available from .decimal LLC.

## 2 Methods

The study involved 11 patients treated for skin lesions located in the corner of the eye in the West Pomeranian Oncology Centre in Szczecin, Poland (Table 1). The patients received a treatment regimen that included the administration of a therapeutic dose of 60 Gy in 30 fractions. Each PTV shape was determined and constructed by a radiation oncologist by adding a 3 mm isotropic margin to the CTV (Clinical Target Volume). Treatment was planned in the Nucletron Oncentra MasterPlan, version 4.3 TPS using a single electron beam of 6 or 9 MeV. The dose distribution was calculated using the Voxel Monte Carlo calculation algorithm^32^ and at least 50,000 number of histories/cm^2^, which equals 50,000 incident electrons/cm^2^. Using computed tomography (CT) scans with 2 mm slice thickness, a bolus individual to each patient was designed in the TPS (reference bolus), which was then produced using a 3D printer — a 3D‐printed bolus. The reference bolus was designed as a water‐equivalent structure having its distal surface conforming to the skin surface; its flat, proximal surface was drawn perpendicular to beam central axis at a location that conformed the 90% dose surface to the distal PTV surface as closely as possible. Once an acceptable dose distribution was confirmed, the bolus was produced using a 3D printer. To demonstrate the advantages of the 3D‐printed bolus over the methods of manual bolus preparation, a paraffin bolus (prepared manually, directly on the patient's body) was also created for each of the 11 patients.

**Table 1:** Detailed planning data for each analyzed patient

Patient number	Disease	Anatomical location of PTV	PTV volume [cm^3^]	Field size dimensions [cm]	Beam energy [MeV]	Maximum depth of PTV [cm]
1	Basal cell carcinoma	Inner canthus	1.00	ø 3.0	6	0.56
2	Basal cell carcinoma	Inner canthus	2.63	ø 3.0	6	1.10
3	Basal cell carcinoma	Outer canthus (lower eyelid)	7.53	6.7 × 3.9	6 and 9	1.80
4	Basal cell carcinoma	Inner canthus	5.99	3.5 × 3.2	6	1.00
5	Basal cell carcinoma	Inner canthus	2.39	ø 3.0	9	2.00
6	Basal cell carcinoma	Inner canthus	1.51	ø 3.2	6	1.00
7	Basal cell carcinoma	Inner canthus	2.14	ø 3.1	6	0.84
8	Squamous cell carcinoma	Inner canthus and lower eyelid	10.00	7.5 × 6.6	9	2.60
9	Squamous cell carcinoma	Inner canthus and lower eyelid	7.51	5.8 × 4.3	9	1.56
10	Basal cell carcinoma	Inner canthus and lower eyelid	1.17	3.9 × 2.5	6	0.70
11	Basal cell carcinoma	Inner canthus	1.30	ø 3.0	6	0.60

Printing a bolus requires the information on the shape of a bolus, which was previously designed using the TPS and stored in DICOM format, to be converted to a format able to be recognized by the 3D printer, namely the STL format.^27^ Each 3D‐printed bolus was prepared using data, stored in DICOM format, containing the precise structural information for each layer of the bolus scanned in the form of a set of vertices, each having a defined 3D location. Using these data and the computer program developed by the authors, individual layers of the bolus were reconstructed and subsequently combined in a triangulation 3D structure, thereby forming a ready‐to‐print bolus in STL format. Nontoxic, biologically inert, ABS (acrylonitrile‐butadiene‐styrene) copolymer with a density 1.05 g/cm^3^ close to the density of soft tissue (1.05 g/cm^3^) and an atomic composition similar to that of the human body was used to print each bolus. Before printing, each bolus was divided into layers of 100‐*μ*m thickness. Boluses were printed in Fused Deposition Modeling (FDM) technology at 100% filling, ensuring uniformity of structure. Total time to print a 3D‐printed bolus (file conversion, file transfer, and 3D printing) was different for the various sizes of the boluses, and ranged from 0.5 to 5 hours (for volumes from 1.4 cm^3^ to 20.0 cm^3^).

To build each paraffin bolus, in TPS, the reference bolus structure was divided into a series of horizontal layers corresponding to the thickness of a single paraffin sheet (1.5 mm). From the shape of layers, the corresponding fields were made, displayed, and manually drawn on the patient's skin during the radiotherapy simulation. On the basis of data obtained from the TPS paper templates were prepared. Next, based on the paper templates, the paraffin sheets were produced. Finally, paraffin bolus was created directly on the patient skin by placing and joining together layers of paraffin (according to the shape drawn on the skin). Total times to prepare paraffin boluses varied with their size, ranging from 0.5 to 2 hours.

**Figure 1:**
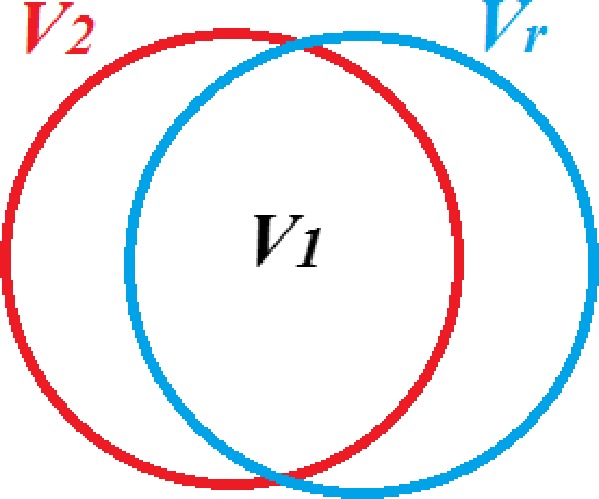
Volumes used to calculate Matching Level (ML) index (Eq. (1)): *V*_*r*_ represents the total volume of the reference model; *V*_*2*_ is the volume of the 3D‐printed or paraffin bolus; and *V*_*1*_ is the volume of the 3D‐printed or paraffin bolus contained in *V*_*r*_.

For quality assurance purposes, a CT scan of each bolus (both 3D‐printed bolus and paraffin bolus) was acquired without the patient. These data were used to evaluate the homogeneity of the boluses. In addition, in TPS, the CT image was downloaded. Using landmark registration option (by selecting three corresponding points on the vertices of images of boluses), the images of the 3D‐printed and paraffin bolus were superimposed onto the CT image of the patient with the reference bolus. The resulting image was used to verify the fit of the 3D‐printed and paraffin bolus with the reference one, and also for simulation of dose distribution when including each bolus (3D‐printed and paraffin) in the treatment plan. To quantitatively measure degree to which the two boluses accurately mapped to the reference one, the authors created a Matching Level Index (ML), based on the coverage index widely used in radiotherapy:

(1) $$ML=\frac{V_{1}}{V_{r}}\cdot \frac{V_{1}}{V_{2}}\cdot 100\%$$

where *V*_1_ is the volume of the 3D‐printed or paraffin bolus contained in a volume of reference bolus; *V*_*r*_ represents the total volume of the reference bolus; and *V*_2_ is the volume of the 3D‐printed or paraffin bolus (Fig. 1). The resulting ML values can range from 0 to 100%, where 100% indicates a perfect fit of the 3D‐printed or paraffin bolus to the reference one.

Finally, fit of both boluses was made directly on the patient skin, during treatment plan evaluation on radiotherapy simulator. To verify the correct position of the bolus on the skin, from TPS, for each patient, projection of the bolus (anterior posterior and lateral) on the patient skin with marked therapeutic field and points corresponding with the points marked on the bolus were printed.

The superimposed images of the 3D‐printed/paraffin bolus with the patient's reference CT was used also for simulation of dose distribution. For this purpose, each bolus was individually outlined and included in the treatment plan. For each bolus, the dose distribution was computed (and subsequently compared with the planned dose distribution for the reference bolus), maintaining the beam geometry and the number of monitor units. In the current study, differences in the dose distributions were assessed using three metrics: minimum, mean, and maximum dose in the PTV, expressed as a percentage dose according to

(2) $$\Delta D=\frac{D_{r}-D_{m}}{D_{r}}\cdot 100\%$$

where *D*_*r*_ is dose for the reference bolus and *D*_*m*_ is dose for the 3D‐printed or paraffin bolus.

## 3 Results

Based on the bolus volume data from the TPS, Table 2 was created. In the case of the 11 patients analyzed, the 3D‐printed bolus was observed to be located in 99–100% of the volume of the reference bolus. Together with a similar volume of the 3D‐printed model (V_2 3D_) and the reference model (V_r_), this resulted in a high Matching Level Index (ML_3D_: 92.5–98.4%). This analysis was also carried out for each paraffin bolus, and the percentage of the paraffin bolus volume contained within the volume of the reference one was in the range of 28.2–99.0%, which combined with the fact that paraffin bolus volume (V_2 paraffin_) was smaller in all patients studied compared to reference bolus (V_r_), resulted in a relatively low Matching Level Index (ML_paraffin_ 23.5–66.6%).

**Table 2:** Results of matching 3D‐printed and paraffin boluses to the reference model for 11 patients. Table contains detailed information: volume of the reference model (V_r_); volumes of the 3D‐printed (V_1 3D_) and paraffin (V_1 paraffin_) boluses contained in the V_r_; volumes of the 3D‐printed (V_2 3D_) and paraffin (V_2 paraffin_) boluses; and the Matching Level Index (Eq. (1)) for 3D‐printed (ML_3D_) and paraffin (ML_paraffin_) boluses. Data distributions of the Matching Level Indexes exhibited normality according to the Shapiro–Wilk test

Patient	V_1 3D_ [cm^3^]	V_1 paraffin_ [cm^3^]	V_r_[cm^3^]	V_2 3D_ [cm^3^]	V_2 paraffin_ [cm^3^]	ML_3D_ [%]	ML_paraffin_ [%]
1	6.8	5.8	7.0	6.9	20.7	96.2	23.5
2	3.5	2.5	3.8	3.5	3.3	92.8	49.9
3	18.5	16.0	20.0	18.5	23.9	92.5	53.6
4	7.0	4.7	7.1	7.1	4.7	98.3	66.6
5	1.4	1.0	1.4	1.4	2.5	94.4	28.5
6	2.3	1.8	2.4	2.3	2.7	94.9	50.3
7	6.1	5.0	6.2	6.1	10	98.4	40.3
8	14.0	10.0	15.0	14.0	14.3	93.3	46.7
9	10.5	7.0	11.0	10.6	10.8	94.5	41.4
10	1.7	1.5	1.8	1.7	2.5	94.4	50.8
11	6.8	5.1	7.0	6.9	6.8	96.2	54.6
				Average		**95.1**	**46.0**
				SD		2.1	10.1

**Figure 2:**
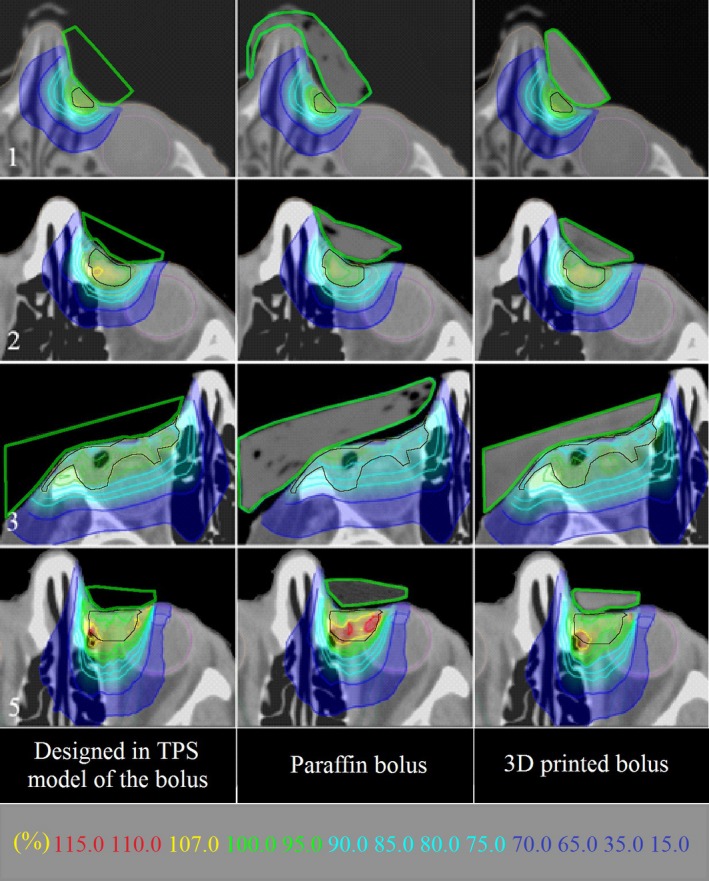
Comparison of dose distributions distal to reference model, paraffin, and 3D‐printed boluses and superimposed on transverse CT scans for select patients (1, 2, 3, 5). Bolus shapes are outlined in green. PTV is outlined in black. Note isodose values in key at bottom of figure.

Using the CT scans of the 11 3D‐printed boluses and the 11 paraffin boluses, the homogeneity of each bolus was examined. The results showed significant heterogeneity in the case of paraffin boluses, resulting from air gaps created during the formation of the bolus (i.e., through joining of the individual layers of the bolus). In the case of the 3D‐printed boluses, which involve the gradual deposition of melted copolymer ‘threads’, no significant heterogeneity was detected. Figure 2 illustrates this for a central CT transverse slice of four patients. Heterogeneities in the structure of the paraffin bolus may result in a change in the dose distribution received by the patient in relation to that without heterogeneity.

Table 3 shows the results of simulation of dose distribution with the inclusion of both boluses (3D‐printed and paraffin) to the treatment plan, showing the percentage differences in the minimum, mean, and maximum dose in PTV coverage in relation to the project with reference bolus. Percentage differences were calculated according the formula (2).

**Table 3:** Percent differences in metrics (near‐minimum, mean, and near‐maximum values of dose distribution within PTV) for 3D‐printed and paraffin boluses relative to those for reference boluses, calculated according to Eq. (2) for all 11 patient treatment plans. Normality of the resulting distribution of data was checked by the Shapiro–Wilk test. When the distribution was normal, the average metric was presented, otherwise the median.*

Patient	Percentage difference in dose distribution *∆D* [%]					
	Near‐minimum PTV dose		Mean PTV dose		Near‐maximum PTV dose	
	3D‐printed bolus	Paraffin bolus	3D‐printed bolus	Paraffin bolus	3D‐printed bolus	Paraffin bolus
1	5.0	9.0	2.5	4.0	2.0	2.5
2	−1.0	3.0	−0.5	2.0	1.5	2.5
3	−3.5	24.0	2.0	5.0	−0.5	−4.0
4	0.0	−2.0	−0.5	−2.0	0.0	−4.0
5	0.5	4.0	0.5	1.5	1.5	8.0
6	0.0	2.0	0.5	2.0	1.5	5.0
7	1.0	4.5	1.0	3.0	2.0	5.0
8	5.0	7.0	2.0	4.0	2.5	3.0
9	−0.5	4.0	−0.5	2.0	2.0	3.0
10	−2.0	7.0	1.5	3.0	−0.5	−4.0
11	0.5	2.0	0.5	2.0	2.0	5.0
Average/median*	0.0*	7.0*	0.8	2.4	1.5*	3.0*
SD	‐	‐	1.1	1.8	‐	‐
Upper quartile	1.0	7.0	‐	‐	2.0	5.0
Lower quartile	−1.0	2.0	‐	‐	0.0	−4.0

The smallest percentage of the difference in the dose in relation to the treatment plan with the reference bolus was obtained for plans including 3D‐printed bolus, where the differences in relation to the reference project did not exceed 5% in the minimum dose and 2.5% in the mean and maximum dose. Results for the paraffin bolus differed significantly from the reference one, with a difference of 24% in the minimum dose, 5% in the mean dose, and 8% in the maximum dose, which is associated with a lack of possibility of precise reproduction of the shape and reduction of heterogeneity in paraffin boluses. The differences in the dose distributions between those for the reference bolus and those for the 3D‐printed and paraffin boluses could also be due to difficulties in the TPS associated with the virtual fitting of both bolus models to the reference bolus shape and patient skin surface.

## 4 Discussion

The use of 3D‐printing technology to produce a bolus can improve the quality of the implemented treatment. Our study has shown that a 3D‐printed bolus has three main advantages over a paraffin bolus manually constructed using paraffin slabs: (1) it is a more precise reconstruction of the reference bolus; (2) it has a better fit to irregular surface of the skin, and (3) it has greater homogeneity.^28^ These advantages provide greater accuracy of dose delivery to the PTV, in accordance with the treatment plan.

Given results of this study and those of others,^27^, ^29^, ^30^ we are confident that 3D‐printed boluses can be used accurately and safely for electron beam therapy.

Authors obviously see the possible limitations. 3D‐printing times for the small boluses used in the present study ranged from 0.5 to 5 hours. Such long printing times could restrict the size of the printed boluses, e.g., those used for chest wall electron therapy.^13^, ^24^, ^28^, ^30^ Of course, the cost of the 3D printer and materials should be taken into consideration.^30^

## 5 Conclusion

In conclusion, 3D printing technology is a viable method for fabricating boluses for small eye lesions and provides boluses superior to ours manually fabricated from paraffin sheets. Additionally, dose distributions using 3D‐printed boluses more closely matched those of the dose plan than those manually fabricated.

## Conflict of interest

The authors declare no conflict of interest.
